# Supplementary material for: Telomere Length, Apoptotic, and Inflammatory Genes: Novel Biomarkers of Gastrointestinal Tract Pathology and Meat Quality Traits in Chickens under Chronic Stress (Gallus gallus domesticus)
Source: Animals (Basel). 2021 Nov 16;11(11):3276. doi: 10.3390/ani11113276 (PMC8614256; doi:10.3390/ani11113276)
Supplement: Supplementary file 1 [file animals-11-03276-s001.zip › animals-1386788-supplementary.pdf]

animals

MDPI

Article

Telomere Length, Apoptotic and Inflammatory genes: Novel Biomarkers of Gastrointestinal Tract Pathology and Meat Quality Traits in Chickens under Chronic Stress (Gallus gallus domesticus)

Kazeem Ajasa Badmus[1,3], Zulkifli Idrus[1,3], Goh Yong Meng[2,3] and Kamalludin Mamat-Hamidi[1,3]*

[1] Department of Animal Science, Universiti Putra Malaysia, Selangor, Malaysia; alqasiminternational@yahoo.com; zulidrus@upm.edu.my; mamath@upm.edu.my
[2] Department of Veterinary Pre-Clinical Science, Universiti Putra Malaysia, Selangor, Malaysia; ymgoh@upm.edu.my
[3] Institute of Tropical Agriculture and Food Security, Universiti Putra Malaysia, Selangor, Malaysia; zulidrus@upm.edu.my; ymgoh@upm.edu.my; mamath@upm.edu.my
* Correspondence: mamath@upm.edu.my

Table S1a: Telomere length (kb/diploid genome) data of the Corticosterone fed chicken and the control at week 4

Trt	TELO Ct	GAPDH Ct	TELO copy no	GAPDH copy no	TELO length
CORT 1	20.09	27.94	1770523	16724	105.8672
2	16.83	25.14	12273205	88216.16	139.1265
3	16.01	25.09	19973847	90875.06	219.7946
4	21.57	32	735128.4	1500.164	490.032
5	14.42	23.94	51353806	179914.3	285.4347
6	16.34	24.96	16418912	98169.32	167.2509
7	17.5	26.35	8244113	42998.29	191.7312
8	16.14	25.28	18489733	81177.89	227.7681
9	17	25	11094549	95864.66	115.7314
10	14.87	23.83	39310042	192060.6	204.6752
CTRL 1	14.74	24.39	42465339	137719.9	308.3457
2	18.41	25.35	4802047	77872.22	61.66573
3	16.14	26.84	18489733	32141.4	575.2623
4	15.29	24.95	30631787	98754.09	310.1825
5	14.23	23.63	57488318	216284.1	265.8001
6	14.98	24.34	36824008	141870.9	259.56
7	17.9	24.98	6500869	97010.15	67.01226
9	16.86	25.2	12056467	85127.96	141.6276
10	16.2	25.46	17842461	72947.45	244.5934

Table S1b: Telomere length (kb/diploid genome) data of the Corticosterone fed chicken and the control at week 6

Trt	TELO Ct	GAPDH Ct	TELO COPY NO	GAPDH COPY NO	TELO LENGTH
CORT 1	19.28	26.44	2864350	40760.3	70.27304
2	16.99	25.7	13577078	63256.7	214.6346
3	15.46	24.92	27690066	100529.4	275.4425
4	16.91	26.62	11703708	36627.7	319.5316
5	15.44	25.13	28020935	88741.64	315.7586
6	15.15	24.48	33287618	130551.8	254.9763
7	14.06	23.55	63595727	226808.4	280.3941
8	16.57	25.8	14322543	59609.22	240.274
9	19.08	27.49	3225614	21847.88	147.6396
CTRL 1	15.53	24.6	29912663	121571.3	246.0504
2	14.68	24.16	44005856	157877.7	278.7338
3	15.53	25.02	26562491	94732.7	280.3941
4	14.18	23.72	59221059	205026.8	288.8454
5	14.15	23.99	60285673	174650.3	345.1794
6	12.4	23.28	1.7E+08	266256.7	640.1674
7	17.79	25.39	6939750	76044.06	91.2596
8	14.98	26.52	36824008	38868.95	947.3888
9	14.36	25.4	53216771	75593.76	703.9836

Table S2: Body weight, feed intake, weight gain FCR and their relative measurement

Weeks	Trt	Body weight	Feed intake	wt gain	FCR	RFI	Rwt	RFCR
4	CORT	967.19	827.78	228.67	3.619976	85.58608	23.64272	0.374278
4	CORT	1064.21	877.78	251.41	3.491428	82.48184	23.6241	0.328077
4	CORT	1120.88	877.78	298.41	2.941523	78.31168	26.62283	0.26243
4	CORT	1071.54	833.33	254.02	3.280568	77.76938	23.70607	0.306155
4	CORT	1047.22	800	265.22	3.016364	76.39274	25.3261	0.288035
4	CTRL	1479.48	944.44	465.37	2.029439	63.83594	31.45497	0.137172
4	CTRL	1574.87	1044.44	509.41	2.050293	66.31912	32.34616	0.130188
4	CTRL	1471.24	950	445.31	2.133345	64.57138	30.26767	0.145003
4	CTRL	1549.47	1027.78	482.07	2.132014	66.33107	31.11193	0.137596
4	CTRL	1471.72	983.3	579.96	1.695462	66.81298	39.40695	0.115203
6	CORT	1503.2	810	242.7	3.337454	53.88505	16.14556	0.222023
6	CORT	1517.74	810	267.54	3.027585	53.36882	17.62753	0.19948
6	CORT	1532.83	900	237.66	3.786922	58.71493	15.50465	0.247054
6	CORT	1409.18	783.33	200.47	3.907467	55.58765	14.226	0.277287
6	CORT	1432.97	808.33	203.97	3.962985	56.40942	14.23407	0.276557
6	CTRL	2229.51	992.86	433.77	2.288909	44.53266	19.45584	0.102664
6	CTRL	2452.13	1058.33	481.99	2.195751	43.15962	19.65597	0.089545
6	CTRL	2319.98	1025	452.69	2.264243	44.18142	19.51267	0.097598
6	CTRL	2472.91	1142.86	475.91	2.40142	46.21519	19.24494	0.097109
6	CTRL	2343.22	1064.29	481.22	2.21165	45.41998	20.5367	0.094385

Table S3: Meat Shear Force of the Corticosterone fed chicken and the control at week 4 and 6

Trt/Week	Shear Force (Kg)	Trt/Week	Shear Force (Kg)	Trt	Shear Force (Kg)
CORT4	1.18806	CORT6	1.51801	Ctrl4	1.78888
CORT4	1.45614	CORT6	2.18586	Ctrl4	1.9475
CORT4	2.7803	CORT6	1.47674	Ctrl4	1.66457
CORT4	1.855	CORT6	1.36011	Ctrl4	1.3726
CORT4	1.80505	CORT6	1.14021	Ctrl4	2.17835
CORT4	1.69962	CORT6	2.0069	Ctrl4	1.13
CORT4	1.25594	CORT6	1.8117	Ctrl4	0.92985
CORT4	1.14192	CORT6	2.35022	Ctrl4	0.9056
CORT4	1.91355	CORT6	1.3005	Ctrl4	0.99973
CORT4	1.15215	CORT6	2.32426	Ctrl4	0.938
CORT4	1.66886	CORT6	2.10703	Ctrl4	0.94158
CORT4	1.79425	CORT6	1.93666		
CORT4	1.174	CORT6	1.03657		
CORT4	0.94465	CORT6	2.52811		
CORT4	0.92176	CORT6	1.17261		
CORT4	0.9757	CORT6	1.75949		
CTRL4	1.45514	Ctrl6	1.29842		
CTRL4	1.76442	Ctrl6	1.19779		
CTRL4	1.56329	Ctrl6	1.63739		
CTRL4	1.4	Ctrl6	1.50084		
CTRL4	1.21438	Ctrl6	1.33812		
CTRL4	1.0189	Ctrl6	1.34821		
CTRL4	1.56493	Ctrl6	1.27833		
CTRL4	1.75748	Ctrl6	1.24886		
CTRL4	1.6999	Ctrl6	1.124		

Table S4a: Small intestinal villi height of CORT fed chicken and control

Weeks	Duod Ht (μm)	Weeks	Jeju Ht (μm)	Weeks	Ileum Ht (μm)
CORT4	794.12	CORT4	299.75	CORT4	574.7
CORT4	671	CORT4	360.01	CORT4	561.05
CORT4	559.82	CORT4	540.53	CORT4	244.56
CORT4	414.07	CORT4	434.7	CORT4	348.78
CORT4	751	CORT4	430.76	CORT4	366.87
CORT4	643.45	CORT4	248.78	CORT4	601.6
CORT4	742.73	CORT4	423.33	CORT4	290
CORT4	489.05	CORT4	287.7	CORT4	283
CTRL4	470.64	CORT4	373.01	CORT4	336.28
CTRL4	442.05	CORT4	520.11	CORT4	292.93
CTRL4	484.17	CORT4	266.51	CORT4	248.78
CTRL4	412.42	CORT4	624.88	CORT4	209.37
CTRL4	645	CORT4	358.84	CORT4	229.82
CTRL4	293.13	CORT4	477.61	CORT4	220.57
CTRL4	487.36	CORT4	438.98	CORT4	248.78
CTRL4	626.98	CORT4	520.11	CORT4	222.9
CTRL4	710.76	CORT4	497.61	CORT4	239.93
CTRL4	426.22	CORT4	624.88	CORT4	257.89
CTRL4	599.62	CORT4	266.51	CORT4	201.05
CTRL4	706.77	CORT4	648.2	CORT4	300.3
CTRL4	546.78	CORT4	600.73	CORT4	340.95
CTRL4	611.64	CORT4	581.2	CORT4	352.12
CTRL4	497.31	CORT4	409.34	CORT4	371.05
CTRL4	411.09	CORT4	619.24	Ctrl4	276.21
CTRL4	377.08	CORT4	358	Ctrl4	388.19
CTRL4	408.68	CORT4	559.78	Ctrl4	303.61

Table S4b: Small intestinal villi height of CORT fed chicken and control

**Weeks**	**Duod Ht** (μm)	**Weeks**	**Jeju Ht** (μm)	**Weeks**	**Ileum Ht** (μm)
CORT6	567.85	CTRL4	357.03	CTRL4	393.2
		CORT4	331.68	CTRL4	347.45
CORT6	446	CORT4	540.59	CTRL4	366.93
CORT6	529.99	CORT4	307.46	CTRL4	325.73
CORT6	313.16	CORT4	307.85	CTRL4	292.58
CORT6	599.63	CTRL4	311.14	CTRL4	187.26
CORT6	255.95	CTRL4	329.69	CTRL4	325.25
CORT6	397.87	CTRL4	365.72	CTRL4	252.42
CORT6	216.45	CTRL4	409.68	CTRL4	238.57
CORT6	355.31	CTRL4	311.46	CTRL4	390.1
CORT6	300.46	CTRL4	399.33	CTRL4	356.23
CORT6	247.72	CTRL4	437.13	CTRL4	445.911
CORT6	809.1	CTRL4	380.94	CTRL4	434.43
CORT6	582.82	CTRL4	321.74	CTRL4	360.98
CORT6	565.68	CTRL4	453.01	CTRL4	204.83
CORT6	549.6	CTRL4	447.78	CTRL4	306.74
CORT6	590.87	CTRL4	249.8	CTRL4	143.25
CORT6	621.68	CTRL4	514.91	CTRL4	199.43
CORT6	606.65	CTRL4	493.31	CTRL4	197.99
CORT6	556.6	CTRL4	579.48	CTRL4	192.07
CORT6	600.32	CTRL4	387.79	CTRL4	304.9
CORT6	587.67	CTRL4	443.66	CTRL4	282.07
CORT6	591.97	CORT6	656.85	CTRL4	296.05
CORT6	573	CORT6	642.44	CTRL4	260.11
CORT6	605.61	CORT6	601	CTRL4	315.34
CORT6	351.12	CORT6	405.45	CORT6	280.5
CORT6	431.79	CORT6	676.15	CORT6	264.97

Table S4c: Small intestinal villi height of CORT fed chicken and control

Weeks	Duod Ht (μm)	Weeks	Jeju Ht (μm)	Weeks	Ileum Ht (μm)
CORT6	249.67	CORT6	728.94	CORT6	283.17
CORT6	788.2	CORT6	661.96	CORT6	393.31
CTRL6	418	CORT6	683.46	CORT6	334.21
CTRL6	802.61	CORT6	739.69	CORT6	318.76
CTRL6	365.25	CORT6	737.63	CORT6	201.42
CTRL6	489.47	CORT6	682.17	CORT6	196.77
CTRL6	422.96	CORT6	509.08	CORT6	231.5
CTRL6	387.81	CORT6	719.19	CORT6	518.14
CTRL6	527.52	CTRL6	683.17	CORT6	629.61
CTRL6	553.75	CTRL6	652.04	CORT6	341.5
CTRL6	303.07	CTRL6	663.73	CORT6	411.39
CTRL6	314.34	CTRL6	619.08	CTRL6	465.14
CTRL6	498.38	CTRL6	570.15	CTRL6	504.15
CTRL6	352.94	CTRL6	612.38	CTRL6	489.28
CTRL6	415.48	CTRL6	555.03	CTRL6	423.49
CTRL6	360.66	CTRL6	513.56	CTRL6	394.15
CTRL6	419.36	CTRL6	346.78	CTRL6	391.23
CTRL6	452.59	CTRL6	607.79	CTRL6	440.16
CTRL6	636.6	CTRL6	538.27	CTRL6	387.64
CTRL6	541.72			CTRL6	366.19
CTRL6	465.14			CTRL6	528.87
CTRL6	504.15			CTRL6	199.98
				CTRL6	377.24
				CTRL6	313.67
				CTRL6	315.22
				CTRL6	324.34
				CTRL6	496.7
				CTRL6	485
				CTRL6	412.03
				CTRL6	264.15
				CTRL6	395.1
				CTRL6	439.98
				CTRL6	496.7

Table S5a: Small intestinal crypt depth of CORT fed chicken and control

Weeks	Duod Cd (µm)	Weeks	Jeju Cd (µm)	Weeks	Ileum Cd (µm)
CORT4	67.36	CORT4	53.64	CORT4	71.23
CORT4	64.27	CORT4	58.67	CORT4	98.82
CORT4	67.36	CORT4	52.25	CORT4	72.07
CORT4	60.31	CORT4	74.24	CORT4	80.8
CORT4	115.16	CORT4	59.3	CORT4	54.22
CORT4	108.97	CORT4	57.57	CORT4	87.91
CORT4	82.93	CORT4	65.31	CORT4	37.57
CORT4	78.37	CORT4	41.08	CORT4	30.98
CORT4	79.65	CORT4	72.28	CORT4	45.36
CORT4	77.35	CORT4	77.31	CORT4	34.36
CTRL4	58.24	CORT4	75.56	CORT4	36.15
CTRL4	53.87	CORT4	32.07	CORT4	45.02
CTRL4	83.79	CORT4	52.69	CORT4	75.2
CTRL4	45.08	CTRL4	64.15	CORT4	87.1
CTRL4	69.27	CTRL4	55.61	CTRL4	43.03
CTRL4	99.12	CTRL4	62.12	CTRL4	71.76
CTRL4	86.15	CTRL4	49.95	CTRL4	49.45
CTRL4	66.17	CTRL4	81.03	CTRL4	39.22
CTRL4	51.53	CTRL4	74.56	CTRL4	86.6
CTRL4	55.06	CTRL4	57.24	CTRL4	49.48
CORT6	64.74	CTRL4	64.97	CTRL4	52.75
CORT6	73.14	CTRL4	73.96	CTRL4	53.6

Table S5b: Small intestinal crypt depth of CORT fed chicken and control

Weeks	Duod Cd (μm)	Weeks	jej Cd (μm)	Weeks	Ileum Cd (μm)
CORT6	60.16	CTRL4	55.26	CTRL4	44.59
CORT6	54.7	CTRL4	67.1	CTRL4	47.16
CORT6	42.56	CTRL4	55.7	CTRL4	32.33
CORT6	54.98	CTRL4	110.93	CTRL4	48.99
CORT6	60.57	CTRL4	85.24	CTRL4	45.69
CORT6	38.15	CTRL4	71	CTRL4	44.63
CORT6	38.05	CORT6	55.11	CTRL4	53.1
CORT6	50.2	CORT6	62.12	CORT6	49.75
CORT6	46.04	CORT6	75.74	CORT6	53.1
CORT6	45.71	CORT6	55.11	CORT6	49
CORT6	75.86	CORT6	78.47	CORT6	46.37
CORT6	61.27	CORT6	40.77	CORT6	31.62
CTRL6	57.81	CORT6	79.65	CORT6	32.03
CTRL6	63.41	CORT6	76.55	CORT6	57.82
CTRL6	47.85	CORT6	73.14	CORT6	48.46
CTRL6	59.41	CORT6	108.66	CORT6	39.12
CTRL6	49.39	CORT6	79.65	CORT6	41.09
CTRL6	58.24	CORT6	41.44	CORT6	33.77
CTRL6	49.39	CORT6	35.79	CORT6	42.63
CTRL6	52.75	CTRL6	65	CORT6	58.25
CTRL6	58.24	CTRL6	57.34	CORT6	43.81
CTRL6	47.85	CTRL6	58.34	CORT6	44.38
CTRL6	59.4	CTRL6	52.72	CORT6	64.18
CTRL6	86.7	CTRL6	54.13	CORT6	60.74
CTRL6	93.36	CTRL6	48.79	CORT6	76.19
CTRL6	104.69	CTRL6	74.6	CTRL6	55.06
CTRL6	91.02	CTRL6	54.13	CTRL6	68.13
CTRL6	110.9	CTRL6	61.31	CTRL6	53.77
CTRL6	115.06	CTRL6	50.31	CTRL6	58.12
CTRL6	39.78			CTRL6	46.12
CTRL6	50.41			CTRL6	63.68
CTRL6	48.99			CTRL6	53.68
				CTRL6	59.15
				CTRL6	63.92
				CTRL6	41.09
				CTRL6	46.46
				CTRL6	74.03

Table S6: Muscle Fibres of CORT fed chicken and control

**Weeks**	**Myofbril (µm)**	**Weeks**	**Myofibril bundles (µm)**
CORT4	53.15	CORT4	301.75
CORT4	49.24	CORT4	452.65
CORT4	52.04	CORT4	472.45
CORT4	57.13	CORT4	145.05
CTRL4	77.41	CORT4	283.55
CTRL4	67.09	CORT4	633.79
CTRL4	67.43	CORT4	374.75
CTRL4	71.85	CORT4	649.11
CTRL4	98.81	CORT4	424.83
CTRL4	64.21	CORT4	417.51
CTRL4	71.34	CORT4	403.19
CTRL4	76.15	CORT4	486
CORT6	74	CORT4	513.17
CORT6	80	CTRL4	409.86
CORT6	79.12	CTRL4	272.85
CORT6	74.97	CTRL4	237.72
CORT6	90	CTRL4	578.66
CTRL6	96.98	CTRL4	802.67
CTRL6	95.72	CTRL4	386.45
CTRL6	97.53	CTRL4	488.31
CTRL6	120.3	CTRL4	1086.06
CTRL6	102.81	CTRL4	856.38
CTRL6	107.51	CTRL4	563.35
CTRL6	77.58	CORT6	230.21
CTRL6	115.11	CORT6	466.92
CTRL6	77	CORT6	353.64
		CORT6	388.02
		CORT6	551.09
		CORT6	665.61
		CTRL6	1014
		CTRL6	687.68
		CTRL6	543.65
		CTRL6	768.35
		CTRL6	856

Table S7: Chemical (Proximate composition) of the meat quality traits of CORT fed chicken and control

Weeks	CP (%)	Weeks	ME (Mj/Kg)	Weeks	EE (%)	Weeks	Moisture (%)	Dm (%)
CORT2	18.47	CORT2	20.778	CORT2	5.666667	CTRL2	75.24603	24.75397
CORT2	16.43	CORT2	20.795	CORT2	6.333333	CTRL2	75.42857	24.57143
CORT2	18.83	CORT2	20.686	CORT2	6	CTRL2	73.3305	26.6695
CORT2	18.36	CORT2	20.815	CORT2	10.73333	CTRL2	71.40381	28.59619
CORT2	18.12	CORT2	21.285	CORT2	5.333333	CTRL2	73.29361	26.70639
CTRL2	18.17	CORT2	19.9	CTRL2	4	CTRL2	75.48677	24.51323
CTRL2	19.16	CORT2	21.58	CTRL2	5	CTRL2	75.52036	24.47964
CTRL2	26.18	CORT2	21.354	CTRL2	7.333333	CTRL2	74.86279	25.13721
CTRL2	15.54	CTRL2	20.781	CTRL2	3.333333	CORT2	72.49825	27.50175
CTRL2	18.08	CTRL2	20.586	CTRL2	6.8	CORT2	73.22835	26.77165
CTRL2	19.11	CTRL2	20.948	CORT4	9.333333	CORT2	74.36811	25.63189
CORT4	19.55	CTRL2	20.436	CORT4	8.666667	CORT2	72.60083	27.39917
CORT4	18.94	CTRL2	21.251	CORT4	9.666667	CORT2	74.05797	25.94203
CORT4	18.79	CTRL2	20.161	CORT4	8	CORT2	72.00957	27.99043
CORT4	18.89	CTRL2	21.062	CORT4	9	CORT2	74.19528	25.80472
CORT4	18.034	CTRL2	20.163	CORT4	5.666667	CORT2	69.51477	30.48523
CORT4	17.81	CORT4	21.598	CORT4	8.566667	CORT2	73.57992	26.42008
CORT4	17.92	CORT4	21.877	CTRL4	8.333333	CTRL4	73.71879	26.28121
CORT4	18.071	CORT4	21.37	CTRL4	13.66667	CTRL4	74.18908	25.81092
CTRL4	19.3	CORT4	21.776	CTRL4	11	CTRL4	75.2057	24.7943
CTRL4	19.26	CORT4	21.617	CTRL4	9	CTRL4	74.51327	25.48673
CTRL4	18.89	CORT4	20.438	CTRL4	5	CTRL4	73.46466	26.53534
CTRL4	19.3	CORT4	20.294	CTRL4	7.733333	CTRL4	74.44332	25.55668
CTRL4	17.32	CORT4	20.842	CTRL4	4.966667	CTRL4	73.96785	26.03215
CTRL4	18.316	CORT4	20.571	CTRL4	5.466667	CTRL4	73.87535	26.12465
CTRL4	19.12	CTRL4	21.347	CTRL4	11.93333	CTRL4	73.87736	26.12264
CTRL4	17.54	CTRL4	22.123			CORT4	71.91235	28.08765
CTRL4	20.189	CTRL4	21.835			CORT4	72.68856	27.31144
		CTRL4	21.57			CORT4	72.2973	27.7027
		CTRL4	21.65			CORT4	74.49306	25.50694
		CTRL4	20.811			CORT4	72.32472	27.67528
		CTRL4	20.294			CORT4	72.34679	27.65321
		CTRL4	20.5			CORT4	71.13772	28.86228
		CTRL4	21.741			CORT4	74.31966	25.68034
		CTRL4	22.805			CORT4	73.3996	26.6004
						CORT4	73.45528	26.54472

Table S8a: Meat Colours of CORT fed chicken and control

Trt	L*	a*	b*
CORT2	52.9	6.73	21.56
CORT2	53.13	6.29	22.65
CORT2	54.96	5.2	21.95
CORT2	59.13	5.82	25.88
CORT2	58.36	5.89	25.83
CORT2	58.28	5.71	26.01
CORT2	54.09	10.11	23.17
CORT2	54.33	8.78	23.36
CORT2	53.69	10.79	23.39
CORT2	51.02	9.68	21.44
CORT2	52.61	8.96	21.39
CORT2	53.3	7.78	21.3
CORT2	56.68	7.51	25.75
CORT2	57.9	7.72	26.48
CORT2	56.6	7.07	23.43
CORT2	54.33	7.87	23.16
CORT2	54.45	7.68	23.29
CORT2	54.23	7.9	23.48
CORT2	53.54	7.9	22.57
CORT2	53.68	7.09	21.88
CORT2	54.57	6.82	22.26
CORT2	54.25	6.61	22.5
CORT2	53.17	7.27	24.55
CORT2	53.42	6.93	24.33
CORT2	52.6	7.49	24.06
CORT2	54.65	8.57	26.11
CORT2	54.19	8.62	26.51
CORT2	53.85	8.79	26.25
CORT2	50.48	7.34	24.55
CORT2	50.59	7.45	24.44
CORT2	50.46	7.15	24.77

Table S8b: Meat Colours of CORT fed chicken and control

Trt	L*	a*	b*
CTRL2	56.42	6.73	21.28
CTRL2	57.09	6.22	21.71
CTRL2	56.69	6.38	22.23
CTRL2	52.69	8.92	21.97
CTRL2	52.61	9.46	22.69
CTRL2	53.48	8.94	23.24
CTRL2	51.87	7.16	21.84
CTRL2	51.58	6.9	21.05
CTRL2	51.51	7.02	21.15
CTRL2	57.39	6.52	20.75
CTRL2	55.89	6.58	20.92
CTRL2	57.4	6.14	20.56
CTRL2	49.36	9.09	21.48
CTRL2	49.96	8.85	22.08
CTRL2	49.6	8.69	21.37
CTRL2	52.1	6.34	21.11
CTRL2	49.43	7.95	21.56
CTRL2	51.59	6.68	20.89
CTRL2	58.34	6.54	23.91
CTRL2	58.51	6.14	23.44
CTRL2	58.3	6.38	24.27
CTRL2	52.75	8.31	22.08
CTRL2	54.18	7.55	22.34
CTRL2	53.34	7.89	22.47

Table S8c: Meat Colours of CORT fed chicken and control

Trt	L*	a*	b*
CORT4	55.75	4.82	24.86
CORT4	55.03	4.61	23.78
CORT4	55.94	4.83	25.76
CORT4	52.88	6.63	19.7
CORT4	52.81	6.51	19.76
CORT4	52.78	6.62	19.99
CORT4	41.03	11.46	19.94
CORT4	40.81	11.21	19.09
CORT4	40.99	11.21	19.69
CORT4	51.01	6.74	18.86
CORT4	51.44	6.49	18.91
CORT4	50.86	6.75	18.91
CORT4	46.58	6.76	18.66
CORT4	46.64	6.71	18.97
CORT4	46.45	6.94	18.52
CORT4	49.72	7.29	18.8
CORT4	49.22	8.43	18.96
CORT4	49.47	7.91	19.26
CORT4	50.47	7.38	26.05
CORT4	51.77	7.36	24.96
CORT4	51.46	7.11	25.89
CORT4	51.2	3.37	15.97
CORT4	52.34	2.71	15.91
CORT4	51.39	3.14	16.18
CORT4	50.17	7.64	22.32
CORT4	49.91	7.4	21.86
CORT4	49.75	7.81	22.74
CORT4	52.86	7.43	22.51
CORT4	52.82	7.34	22.35
CORT4	53.04	7.82	22.85

Table S8d: Meat Colours of CORT fed chicken and control

Trt	L*	a*	b*
CTRL4	50.08	6.52	16.17
CTRL4	49.62	6.46	16.18
CTRL4	49.52	6.5	16.42
CTRL4	59.3	3.74	18.67
CTRL4	59.01	3.61	18.44
CTRL4	59.18	3.36	18.26
CTRL4	53.27	8.14	22.16
CTRL4	51.91	9.13	22.79
CTRL4	53.69	7.69	22.06
CTRL4	59.71	4.38	20.87
CTRL4	58.99	4.73	20.73
CTRL4	59.66	4.46	20.65
CTRL4	59.19	2.71	17.51
CTRL4	58.94	2.11	16.13
CTRL4	49.45	4.45	19.22
CTRL4	50.6	4.43	19.49
CTRL4	51.7	4.07	19.68
CTRL4	53.17	4.83	20.31
CTRL4	53.31	5.01	20.12
CTRL4	53.27	4.67	19.85
CTRL4	56.51	5.11	23.44
CTRL4	57.79	5	23.52
CTRL4	57.5	5.36	22.93
CTRL4	54.22	4.75	19.35
CTRL4	54.05	4.8	19.42
CTRL4	54.12	4.68	19.64
CTRL4	59.35	3.01	19.49
CTRL4	60.28	2.92	19.61
CTRL4	58.83	3.27	19.58
CTRL4	54.89	7.61	25
CTRL4	54.51	8.04	26.34
CTRL4	54.96	7.73	25.58
CTRL4	54.42	7.67	25.51
CTRL4	51.9	7.03	23.31
CTRL4	51.4	7.37	24.32
CTRL4	51.97	6.74	23.62

Table S9: pH, Drip loss and cooking loss of of CORT fed chicken and control

Trt	pH	Trt	DL (%)	Trt	CL (%)
CORT2	5.54	CORT2	2.937788	CORT2	24.16174
CORT2	5.27	CORT2	4.433498	CORT2	26.23815
CORT2	5.55	CORT2	4.226543	CORT2	22.22222
CORT2	5.41	CORT2	1.777778	CORT2	31.36027
CORT2	5.5	CORT2	2.839117	CORT2	18.29871
CORT2	5.47	CORT2	2.702703	CORT2	23.91304
CORT2	5.41	CORT2	3.324808	CORT2	23.94247
CORT2	5.47	CORT2	3.162055	CORT2	26.33567
CORT2	5.58	CORT2	2.614897	CORT2	28.30937
CORT	5.43	CTRL2	2.351157	CORT2	22.73442
CTRL2	5.62	CTRL2	2.036324	CTRL2	32.72942
CTRL2	5.65	CTRL2	1.188061	CTRL2	49.91658
CTRL2	5.83	CTRL2	1.851229	CTRL2	33.43492
CTRL2	5.69	CTRL2	1.896463	CTRL2	28.92243
CTRL2	5.64	CTRL2	2.261307	CTRL2	16.22003
CTRL2	5.64	CTRL2	2.581095	CTRL2	24.06554
CTRL2	5.9	CTRL2	1.417482	CTRL2	31.96203
CTRL2	5.66	CORT4	4.311927	CTRL2	29.16796
CTRL2	5.63	CORT4	8.693772	CTRL2	41.16248
CTRL2	5.45	CORT4	4.774436	CTRL2	20.67019
CORT4	6.42	CORT4	6.219081	CORT4	26.00281
CORT4	6.23	CORT4	4.620231	CORT4	26.31579
CORT4	6.34	CORT4	4.224626	CORT4	27.6176
CORT4	6.06	CORT4	5.127082	CORT4	25.30928
CORT4	6.41	CORT4	6.132542	CORT4	48.50338
CORT4	5.93	CORT4	4.149835	CORT4	27.96185
CORT4	6	CORT4	3.317811	CORT4	19.25134
CORT4	6.37	CTRL4	3.675937	CORT4	29.7434
CORT4	5.92	CTRL4	3.65	CORT4	18.3278
CORT4	5.63	CTRL4	2.868104	CORT4	28.01095
CTRL4	5.63	CTRL4	3.570555	CTRL4	26.39849
CTRL4	5.67	CTRL4	4.677966	CTRL4	46.63262
CTRL4	5.49	CTRL4	2.61661	CTRL4	31.59443
CTRL4	5.92	CTRL4	3.649794	CTRL4	29.00552
CTRL4	5.72	CTRL4	3.268156	CTRL4	29.6671
CTRL4	6.01	CTRL4	3.60151	CTRL4	20.53649
CTRL4	5.63	CTRL4	4.157489	CTRL4	26.70988
CTRL4	6.02			CTRL4	46.07247
CTRL4	6.01			CTRL4	32.29116
CTRL4	6.02			CTRL4	22.48791

Table S10a: Gene expression of *COX6A1* of Muscle at week 4 of CORT fed chicken and control

Trt	*GAPDH* Av.Ct RG	*B-actin* Av.Ct RG	Ref. av	*COX6A1* Av.Ct Test	2-ΔΔCt
CTRL	32.69	29.3	30.94862	31.6	1
CTRL	34.63	36.06	35.33777	37.72	1
CTRL	26.56	30.22	28.33096	36.21	1
CTRL	29.64	36.68	32.97264	32.62	1
CTRL	31.18	30.99	31.08485	33.29	1
CTRL	25.75	30.22	27.89561	23.24	1
CTRL	37.3	34.15	35.69026	36.97	1
CORT	26.94	32.26	29.48024	31.46	0.642264
CORT	29.5	29	29.24893	32.42	0.281251
CORT	29.72	28.1	28.89865	33.35	0.115795
CORT	29.82	32	30.89078	30.38	3.609441
CORT	32.48	28.1	30.21073	33.24	0.310298
CORT	31.3	31.24	31.26999	34	0.381826
CORT	29.82	33.05	31.39349	31.15	2.999011

Tabel S10b: Gene expression of *UCP3* of Muscle at week 4 of CORT fed chicken and control

Trt	GAPDH Av.Ct RG	B-actin Av.Ct RG	Ref. av	UCP3 Av.Ct Test	2-ΔΔCt
CTRL	34.63	30.22	32.34994	30.03	1
CTRL	26.56	36.68	31.21251	21.42	1
CTRL	29.64	30.99	30.30748	22.21	1
CTRL	31.18	30.22	30.69625	21.37	1
CTRL	25.75	34.15	29.65405	24.75	1
CORT	29.5	29	29.24893	22.26	1.07098
CORT	29.72	28.1	28.89865	22.08	0.951747
CORT	29.82	32	30.89078	24.7	0.615903
CORT	25.03	29.73	27.27896	21.77	0.383943
CORT	32.48	28.1	30.21073	25.08	0.295397
CORT	31.3	31.24	31.26999	27	0.162666

Table S10c: Gene expression of *COX6A1* of Muscle at week 6 of CORT fed chicken and control

Trt	*GAPDH* Av.Ct RG	*B-actin* Av.Ct RG	Ref. av	*COX6A1* Av.Ct Test	2-ΔΔCt
CTRL	26.06	27.63	26.83352008	30.06	1
CTRL	35.68	36.41	36.04315192	35.11	1
CTRL	29.34	28.36	28.84583852	30.64	1
CTRL	26.29	27.93	27.09759583	31.04	1
CTRL	28.94	30.51	29.71463276	31.73	1
CTRL	26.57	28.75	27.63851479	31.35	1
CTRL	28.93	27.93	28.4256029	27.75	1
CORT	28.72	30.69	29.6886645	32.06	0.706453
CORT	28.6	30.28	29.42801386	35.05	0.074223
CORT	26.7	28.64	27.6529926	30.89	0.387695
CORT	24.31	27.72	25.95906778	31.47	0.080162
CORT	24.78	30.18	27.3470364	34.06	0.034844
CORT	25.62	28.27	26.91240235	31.35	0.168685
CORT	25.84	28.62	27.19449944	30.8	0.300305
CORT	26.55	28.37	27.44491756	29.83	0.699753

Table S10d: Gene expression (fold change) of *UCP3* of Muscle at week 6 of CORT fed chicken and control

Trt	*GAPDH* Av.Ct RG	*B-actin* Av.Ct RG	Ref.av	*UCP3* Av.Ct Test	2-ΔΔCt
CTRL	26.06	27.63	26.83352008	23.1	1
CTRL	35.68	36.41	36.04315192	36.01	1
CTRL	26.29	28.36	27.30539141	22.03	1
CTRL	28.94	27.93	28.4305153	23.84	1
CTRL	22.16	30.51	26.00195377	23.14	1
CTRL	26.57	28.75	27.63851479	23.03	1
CTRL	28.93	27.93	28.4256029	23.28	1
CORT	28.72	30.69	29.6886645	26.03	0.938653
CORT	34.92	36.5	35.70126048	32.91	0.514506
CORT	33	36.22	34.57253245	34.33	0.087932
CORT	28.6	30.28	29.42801386	24.46	2.326262
CORT	26.7	28.64	27.6529926	21.38	5.747731
CORT	24.31	27.72	25.95906778	21.21	1.998708
CORT	24.78	30.18	27.3470364	21.24	5.123169
CORT	25.62	28.27	26.91240235	21.12	4.119309
CORT	25.84	28.62	27.19449944	23.3	1.105347
CORT	26.55	28.37	27.44491756	22.98	1.641389

Table S10e: Gene expression (fold change) of COX6A1 of Liver at week 4 of CORT fed chicken and control

**Trt**	*GAPDH* **Av.Ct RG**	*B-actin* **Av.Ct RG**	**Ref.av**	*COX6A1* **Av.Ct Test**	**2-ΔΔCt**
CTRL	27.09	28.25	27.66392	26.56	1
CTRL	23.66	23.32	23.48938	25.4	1
CTRL	24.2	23.85	24.02436	26.58	1
CTRL	25.29	24.98	25.13452	28.71	1
CTRL	25.57	25.38	25.47482	25.41	1
CTRL	23.96	23.88	23.91997	27.67	1
CTRL	28.45	29.76	29.09763	31.99	1
CTRL	26.48	26.27	26.37479	30.54	1
CTRL	27.68	28.25	27.96355	27.38	1
CTRL	26.9	26.88	26.89	27.65	1
CORT	28.57	30.02	29.28603	29.16	3.747757
CORT	24.86	25.34	25.09885	26.21	1.589808
CORT	29.46	29.59	29.52493	29.47	3.567537
CORT	25.25	24.8	25.02399	25.33	2.777888
CORT	28.67	28.21	28.43907	27.51	6.539
CORT	26.88	27.78	27.3263	27.79	2.490258
CORT	27.07	26.85	26.95978	27.39	2.548725
CORT	26.12	25.4	25.75748	24.84	6.486698
CORT	26.97	29.98	28.4352	27.5	6.566843
CORT	29.52	29.77	29.64474	28.85	5.957621

Table S10f: Gene expression (fold change) of *UCP3* of Liver at week 4 of CORT fed chicken and control

**Trt**	***GAPDH* Av.Ct RG**	***B-actin* Av.Ct RG**	**Ref.av**	***UCP3* Av.Ct Test**	**2-ΔΔCt**
CTRL	27.09	28.25	27.66392	26.75	1
CTRL	23.66	23.32	23.48938	27.73	1
CTRL	24.2	23.85	24.02436	23.89	1
CTRL	25.29	24.98	25.13452	24.82	1
CTRL	25.57	25.38	25.47482	25.28	1
CTRL	23.96	23.88	23.91997	24.22	1
CTRL	28.45	29.76	29.09763	27.35	1
CTRL	26.48	26.27	26.37479	38.53	1
CTRL	27.68	28.25	27.96355	27.39	1
CTRL	26.9	26.88	26.89	27.44	1
CORT	28.57	30.02	29.28603	29.64	1.980723
CORT	24.86	25.34	25.09885	24.44	3.99682
CORT	29.46	29.59	29.52493	28.83	4.098024
CORT	25.25	24.8	25.02399	24.86	2.836257
CORT	28.67	28.21	28.43907	27.77	4.025226
CORT	26.88	27.78	27.3263	27.77	1.86128
CORT	27.07	26.85	26.95978	27.7	1.515481
CORT	26.12	25.4	25.75748	25.48	3.068395
CORT	26.97	29.98	28.4352	27.66	4.332501
CORT	29.52	29.77	29.64474	28.34	6.253815

Table S10g: Gene expression (fold change) of *COX6A1* of Liver at week 6 of CORT fed chicken and control

Trt	*GAPDH* Av.Ct RG	*B-actin* Av.Ct RG	Ref.	*COX6A1* Av.Ct Test	2-ΔΔCt
CTRL	27.17	28.22	27.69002	31.33	1
CTRL	24.72	24.89	24.80485	24.65	1
CTRL	26.92	26.92	26.92	27.98	1
CTRL	25.88	26.92	26.39488	29.95	1
CTRL	25.19	25.55	25.36936	23.63	1
CTRL	26.05	27.08	26.56001	27.52	1
CTRL	27.53	27.69	27.60988	29.48	1
CTRL	24.86	29.29	26.98424	27.18	1
CTRL	26.27	33.25	29.55465	26.73	1
CTRL	27.69	24.42	26.00365	25.33	1
CORT	27.68	28.54	28.10671	30.53	0.280624
CORT	27.33	28.17	27.74682	28.78	0.735512
CORT	27.37	27.7	27.53451	27.09	2.048412
CORT	26.34	27.57	26.94798	27.23	1.237976
CORT	26.83	26.62	26.72479	26.95	1.287698
CORT	26.77	26.62	26.69489	27.18	1.075416
CORT	26.46	27.27	26.86195	27.68	0.853786
CORT	26.62	26.72	26.66995	27.63	0.773757

Table S10h: Gene expression (fold change) of *UCP3* of Liver at week 6 of CORT fed chicken and control

**Trt**	***GAPDH* Av.Ct RG**	***B-actin* Av.Ct RG**	**Ref.av**	***UCP3* Av.Ct Test**	**2-ΔΔCt**
CTRL	27.17	28.22	27.69002	28.34	1
CTRL	24.72	24.89	24.80485	23.9	1
CTRL	26.92	26.92	26.92	25.99	1
CTRL	25.88	26.92	26.39488	27.07	1
CTRL	25.19	25.55	25.36936	25.12	1
CTRL	26.05	27.08	26.56001	27.42	1
CTRL	27.53	27.69	27.60988	26.59	1
CTRL	24.86	29.29	26.98424	26.09	1
CTRL	26.27	33.25	29.55465	27.66	1
CTRL	27.69	24.42	26.00365	27.98	1
CORT	27.68	28.54	28.10671	27.5	1.353515
CORT	27.33	28.17	27.74682	27.54	1.025851
CORT	27.37	27.7	27.53451	27.21	1.11304
CORT	26.34	27.57	26.94798	27.27	0.71103
CORT	26.83	26.62	26.72479	25.86	1.618653
CORT	26.77	26.62	26.69489	26.39	1.098013
CORT	26.67	27.27	26.96833	27.32	0.696566
CORT	27.15	26.72	26.93414	26.9	0.910128
CORT	26.46	26.88	26.66917	27.4	0.53558
CORT	26.62	27.86	27.23294	26.21	1.806182

Table S10i: Gene expression (fold change) of *SAAL1* of Liver at week 4 of CORT fed chicken and control

**Trt**	***GAPDG* Av.Ct RG**	***B-actin* Av.Ct RG**	**Ref.av**	***SAAL1* Av.Ct Test**	**2-ΔΔCt**
CTRL	27.09	28.25	27.66392	26.76	1
CTRL	23.66	23.32	23.48938	23.8	1
CTRL	24.2	23.85	24.02436	23.88	1
CTRL	25.29	24.98	25.13452	25.08	1
CTRL	25.57	25.38	25.47482	25.08	1
CTRL	23.96	23.88	23.91997	23.75	1
CTRL	28.45	29.76	29.09763	22.54	1
CTRL	26.48	26.27	26.37479	29.43	1
CTRL	27.68	28.25	27.96355	27.85	1
CTRL	26.9	26.88	26.89	26.5	1
CORT	28.57	30.02	29.28603	28.67	1.054111
CORT	24.86	25.34	25.09885	24.39	1.124164
CORT	25.25	24.8	25.02399	25.68	0.43648
CORT	28.67	28.21	28.43907	26.92	1.971194
CORT	26.88	27.78	27.3263	27.18	0.761172
CORT	27.07	26.85	26.95978	27.22	0.57426
CORT	26.12	25.4	25.75748	25.39	0.887294
CORT	26.97	29.98	28.4352	26.57	2.505676
CORT	29.52	29.77	29.64474	27.59	2.857466

Table S10j: Gene expression (fold change) of *SAAL1* of Liver at week 6 of CORT fed chicken and control

Trt	*GAPDH* Av.Ct RG	*B-actin* Av.Ct RG	Ref.av.	*SAAL1* Av.Ct Test	2-ΔΔCt
CTRL	27.17	28.22	27.69002	27.26	1
CTRL	24.72	24.89	24.80485	25.19	1
CTRL	26.92	26.92	26.92	26.8	1
CTRL	25.88	26.92	26.39488	27.54	1
CTRL	25.19	25.55	25.36936	26	1
CTRL	26.05	27.08	26.56001	28.04	1
CTRL	24.86	27.69	26.23687	28.88	1
CTRL	26.27	29.29	27.73893	26.57	1
CTRL	27.69	33.25	30.34292	28.99	1
CORT	27.68	28.54	28.10671	27.32	2.214086
CORT	27.33	28.17	27.74682	27.74	1.289509
CORT	27.37	27.7	27.53451	27.1	1.734483
CORT	26.34	27.57	26.94798	26.6	1.633519
CORT	26.83	26.62	26.72479	26.42	1.585342
CORT	26.77	26.62	26.69489	27.45	0.760434
CORT	26.67	27.27	26.96833	25.4	3.806147
CORT	27.15	26.72	26.93414	27.08	1.160014
CORT	26.62	26.88	26.74968	28.18	0.476215

Table S10k: Gene expression (fold change) of *CRP* of Liver at week 4 of CORT fed chicken and control

Trt	*GAPDG* Av.Ct RG	*B-actin* Av.Ct RG	Ref.av	Av.Ct Test	2-ΔΔCt
1	27.09	28.25	27.66392	25.77	
CTRL	23.66	23.32	23.48938	22.15	1
CTRL	24.2	23.85	24.02436	21.73	1
CTRL	25.29	24.98	25.13452	22.97	1
CTRL	25.57	25.38	25.47482	23.22	1
CTRL	23.96	23.88	23.91997	21.74	1
CTRL	28.45	29.76	29.09763	28.95	1
CTRL	26.48	26.27	26.37479	25.58	1
CTRL	27.68	28.25	27.96355	25.91	1
CTRL	26.9	26.88	26.89	25.36	1
CORT	28.57	30.02	29.28603	27.63	0.990362
CORT	24.86	25.34	25.09885	25.28	0.277172
CORT	29.46	29.59	29.52493	26.39	2.760498
CORT	25.25	24.8	25.02399	23.64	0.820166
CORT	26.88	27.78	27.3263	25.14	1.430277
CORT	27.07	26.85	26.95978	24.99	1.230953
CORT	26.12	25.4	25.75748	24.96	0.546194
CORT	29.52	29.98	29.74911	27.45	1.546612

Table S10l: Gene expression (fold change) of *CRP* of Liver at week 6 of CORT fed chicken and control

Trt	*GAPDH* Av.Ct RG	*B-actin* Av.Ct RG	Ref.av	Av.Ct Test	2-ΔΔCt
CTRL	27.17	28.22	27.69002	28.45	1
CTRL	24.72	24.89	24.80485	26.3	1
CTRL	26.92	26.92	26.92	26.85	1
CTRL	25.88	26.92	26.39488	26.36	1
CTRL	25.19	25.55	25.36936	24.77	1
CTRL	26.05	27.08	26.56001	27.87	1
CTRL	27.53	27.69	27.60988	27.54	1
CTRL	26.27	29.29	27.73893	29.38	1
CTRL	27.69	33.25	30.34292	31.2	1
CORT	27.33	28.54	27.92845	28.6	0.94504
CORT	27.37	28.17	27.76712	26.87	2.803286
CORT	26.77	27.57	27.16706	26.02	3.333541
CORT	26.67	26.62	26.64499	30.58	0.098413
CORT	26.46	26.62	26.53988	31.01	0.067915
CORT	26.62	27.27	26.94304	29.6	0.238662

Table S10m: Gene expression (fold change) of *COX6A1* of Heart at week 6 for the CORT fed chicken and control

Trt	*GAPDH* Av.Ct RG	*B.actin* Av.Ct RG	Ref.av.	*COX6A1* Av.Ct Test	2-ΔΔCt
CTRL	26.13	28.19	27.14046	27.11	1
CTRL	26.61	28.91	27.73617	32.29	1
CTRL	30.44	30.1	30.26952	30.96	1
CTRL	31.5	32.02	31.75894	29.56	1
CORT	30.73	32.95	31.82065	31.1	2.771459
CORT	29.43	30.49	29.95531	30.8	0.936475
CORT	29.99	30.45	30.21912	29.5	2.768539
CORT	29.16	30.79	29.96392	28.93	3.443602
CORT	27.9	29.36	28.62069	30.14	0.586699
CORT	26.98	27.58	27.27835	26.21	3.526777

Table S10n: Gene expression (fold change) of *UCP3* of Heart at week 4 for the CORT fed chicken and control

Trt	*B-actin* Av.Ct RG	*GAPDH* Av.Ct RG	Ref. av.	*UCP3* Av.Ct Test	2-ΔΔCt
CTRL	30.74	29.66	30.19517	30.56	1
CTRL	29.5	29.02	29.25902	30.23	1
CTRL	29.49	29.21	29.34967	29.41	1
CTRL	35.86	34.19	35.01505	35.3	1
CTRL	36.01	39.29	37.61426	35.27	1
CTRL	28.91	27.8	28.34957	27.18	1
CTRL	32.59	27.63	30.00769	32.44	1
CORT	36.82	32.82	34.76251	34.75	1.07067
CORT	28.51	35.11	31.63836	28.38	10.15673
CORT	26.84	27.5	27.168	26.43	1.770302
CORT	28.36	26.18	27.24821	28.07	0.600488
CORT	27.55	33.3	30.28886	27.18	9.1569
CORT	32.64	33.87	33.24931	31.73	3.042617

Table S10o: Gene expression (fold change) of *COX6A1* of Heart at week 4 of CORT fed chicken and control

TRt	*B-actin* Av.Ct RG	*GAPDH* Av.Ct RG	Ref. av.	*COX6A1* Av.Ct Test	2-ΔΔCt
CTRL	30.74	29.66	30.19517	28.84	1
CTRL	29.5	29.02	29.25902	35.13	1
CTRL	29.49	29.21	29.34967	33.94	1
CTRL	35.86	34.19	35.01505	34.51	1
CTRL	36.01	39.29	37.61426	35.56	1
CTRL	28.91	27.8	28.34957	27.88	1
CTRL	32.59	27.63	30.00769	29.8	1
CORT	33.98	34.78	34.37767	34.86	1.281357
CORT	36.82	32.82	34.76251	33.93	3.187697
CORT	26.84	27.5	27.168	31.17	0.111723
CORT	28.36	26.18	27.24821	27.61	1.393012
CORT	36.39	27.54	31.65724	38.05	0.021304

Table S10p: Gene expression (fold change) of *UCP3* of Heart at week 6 of CORT fed chicken and control

**TRt**	*GAPDH* **Av.Ct RG**	*B.actin* **Av.Ct RG**	**Ref.av**	*UCP3* **Av.Ct Test**	**2-ΔΔCt**
CTRL	26.13	28.19	27.14046	28.38	1
CTRL	26.61	28.91	27.73617	29.39	1
CTRL	30.44	30.1	30.26952	29.78	1
CTRL	31.5	32.02	31.75894	30.45	1
CORT	30.73	32.95	31.82065	30.75	2.532647
CORT	29.43	30.49	29.95531	31.04	0.568531
CORT	29.99	30.45	30.21912	31.06	0.673208
CORT	29.16	30.79	29.96392	30.06	1.128118
CORT	27.9	29.36	28.62069	29.08	0.877026
CORT	26.98	27.58	27.27835	30.15	0.16475

Table S10q: Gene expression of *SAAL1* of hypothalamus at week 4 of CORT fed chicken and control

**TRt**	*GAPDH* **Av.Ct RG**	*B-Actin* **Av.Ct RG**	**Ref. av**	*SAAL1* **Av.Ct Test**	**2-ΔΔCt**
CTRL	27.75	28.21	27.97905	26.89	1
CTRL	27.56	28.07	27.81383	26.82	1
CTRL	27.33	27.63	27.47959	26.42	1
CTRL	27.29	28.26	27.77077	26.75	1
CTRL	26.78	27.5	27.13761	26.45	1
CTRL	26.25	26.97	26.60756	25.49	1
CTRL	22.83	38.48	29.63947	27.68	1
CTRL	26.41	35.03	30.41615	26.63	1
CTRL	30.63	28.5	29.54581	26.84	1
CTRL	26.75	28.82	27.76572	26.08	1
CORT	27.12	28.2	27.65473	26.85	0.572254
CORT	26.38	27.52	26.94397	26.09	0.592124
CORT	26.3	27.48	26.88353	26.29	0.494323
CORT	26.94	26.74	26.83981	26.33	0.466456
CORT	27.4	28.16	27.7774	26.86	0.618738
CORT	26.77	28.62	27.67955	26.26	0.876331
CORT	26.02	27.82	26.90495	25.78	0.714473
CORT	28.18	28.15	28.165	27.53	0.508738

Table S10r: Gene expression (fold change) of *CRP* of hypothalamus at week 4 of CORT fed chicken and control

	GAPDH Av.Ct RG	*B-Actin* Av.Ct RG	Ref.av	Av.Ct Test	2-ΔΔCt
CTRL	27.75	28.21	27.97905	25.77	1
CTRL	27.56	28.07	27.81383	25.3	1
CTRL	27.33	27.63	27.47959	24.6	1
CTRL	27.29	28.26	27.77077	24.35	1
CTRL	26.78	27.5	27.13761	23.61	1
CTRL	26.25	26.97	26.60756	23.35	1
CTRL	26.41	38.48	31.87878	29.96	1
CTRL	30.63	35.03	32.7562	32.65	1
CTRL	26.75	28.5	27.61114	30.06	1
CORT	29.18	34.39	31.67807	27.08	6.355789
CORT	27.12	28.2	27.65473	25.73	0.996353
CORT	26.38	27.52	26.94397	23.73	2.435084
CORT	26.3	27.48	26.88353	26.37	0.374627
CORT	27.4	26.74	27.06799	24.29	1.799989
CORT	26.77	28.16	27.45621	23.56	3.90739
CORT	26.02	28.62	27.28905	24.51	1.801318
CORT	28.18	27.82	27.99942	26.37	0.811927

Table S10s: Gene expression (fold change) of *SAAL1* of hypothalamus at week 6 of CORT fed chicken and control

	GAPDH Av.Ct HG	*B-Actin* Av.Ct HG	Ref.av	*SAAL1* Av.Ct Test	2-ΔΔCt
CTRL	26.24	27.03	26.63207	25.81	1
CTRL	26.63	26.91	26.76963	26.25	1
CTRL	26.84	27.28	27.05911	26.84	1
CTRL	26.97	26.83	26.89991	26.58	1
CTRL	26.79	26.67	26.72993	27	1
CTRL	26.51	27.09	26.79843	27.07	1
CTRL	26.3	29.78	27.98596	29.82	1
CTRL	32.11	28.21	30.0969	27.72	1
CTRL	27.53	29.51	28.50281	29	1
CORT	27.09	28.24	27.65902	27.14	1.291479
CORT	26.8	28.25	27.51545	26.57	1.735619
CORT	26.81	27.92	27.35937	26.38	1.776911
CORT	26.77	27.67	27.21628	26	2.094027
CORT	25.17	26.68	25.914	25.2	1.478366
CORT	27.09	27.68	27.38341	26.42	1.757362
CORT	27.04	27.26	27.14978	27.04	0.972505
CORT	26.57	27.06	26.81388	27.21	0.68486
CORT	27.67	27.64	27.655	28.89	0.382888
CORT	27.46	27.73	27.59467	27.9	0.729343

Table S10t: Gene expression (fold change) of *CRP* of hypothalamus at week 6 of CORT fed chicken and control

	GAPDH Av.Ct HG	*B-Actin* Av.Ct HG	Ref.av	Av.Ct Test	2-ΔΔCt
CTRL	26.24	27.03	26.63207	26.83	1
CTRL	26.63	26.91	26.76963	26.43	1
CTRL	26.84	27.28	27.05911	25.89	1
CTRL	26.97	26.83	26.89991	25.1	1
CTRL	26.79	26.67	26.72993	25.62	1
CTRL	26.51	27.09	26.79843	26.11	1
CTRL	26.3	29.78	27.98596	26.6	1
CTRL	32.11	28.21	30.0969	29.49	1
CTRL	27.53	29.51	28.50281	28.36	1
CORT	27.09	28.24	27.65902	28.02	0.453453
CORT	26.81	28.25	27.52058	26.75	0.993494
CORT	26.77	27.92	27.33895	25.71	1.801194
CORT	25.17	27.67	26.39041	25.01	1.516151
CORT	27.09	26.68	26.88422	26.39	0.820297
CORT	27.04	27.68	27.35813	26.85	0.828244
CORT	26.57	27.26	26.91279	25.79	1.268206
CORT	27.67	27.06	27.3633	28.36	0.29185

LIST OF ABBREVIATIONS

a*	Meat redness
b*	Meat yellowness
β-Actin	Beta actin
CORT	Corticosterone
CTRL	Control
CL	Cooking loss
COX6A1	Cytochrome C oxidase 1
CRP	C-reactive protein
DL	Drip Loss
GAPDH	Glyceraldehyde 3-phosphate dehydrogenase
L*	Meat Lightness
qPCR	Quantitative polymerase chain reaction
SAAL1	Serum amyloid alpha 1
Kg	Kilogram
Kb	Kilobase
µl	Microlitre
µm	Micromolar

UCP3	Uncoupling protein P
ΔCt	Change in threshold cycle
Trt	Treatment
Jeju	Jejunum
Duod	Duodenum
Ht	Height
CD	Crypt depth
wt	Weight
FCR	Feed conversion ratio
RFI	Relative feed intake
Rwt	Relative weight gain
RFCR	Relative FCR
TELO	Telomere
S	Supplementary data
Mj/Kg	Megajoule
DM	Dry matter
EE	Ether extract
ME	Metabolizable energy
CP	Crude protein
pH	Degree of acidity and alkalinity
Av.Ct	Average threshold cycle
Ref. av.	Reference genes average using geometry means
